# The association of anxiety disorders and depression with facial scarring: population-based, data linkage, matched cohort analysis of 358 158 patients

**DOI:** 10.1192/bjo.2023.547

**Published:** 2023-11-15

**Authors:** John A. G. Gibson, Thomas D. Dobbs, Rowena Griffiths, Jiao Song, Ashley Akbari, Owen Bodger, Hayley A. Hutchings, Ronan A. Lyons, Ann John, Iain S. Whitaker

**Affiliations:** Reconstructive Surgery & Regenerative Medicine Research Centre, Institute of Life Science, Swansea University Medical School, UK; and The Welsh Centre for Burns and Plastic Surgery, Morriston Hospital, UK; Population Data Science, Swansea University Medical School, Faculty of Medicine, Health & Life Science, Swansea University, UK; Population Data Science, Swansea University Medical School, Faculty of Medicine, Health & Life Science, Swansea University, UK; and Patient and Population Health and Informatics Research, Swansea University Medical School, Faculty of Medicine, Health & Life Science, Swansea University, UK; Patient and Population Health and Informatics Research, Swansea University Medical School, Faculty of Medicine, Health & Life Science, Swansea University, UK

**Keywords:** Anxiety or fear-related disorders, depressive disorders, epidemiology, risk assessment, trauma and stressor-related disorders

## Abstract

**Background:**

Estimates suggest that 1 in 100 people in the UK live with facial scarring. Despite this incidence, psychological support is limited.

**Aims:**

The aim of this study was to strengthen the case for improving such support by determining the incidence and risk factors for anxiety and depression disorders in patients with facial scarring.

**Method:**

A matched cohort study was performed. Patients were identified via secondary care data sources, using clinical codes for conditions resulting in facial scarring. A diagnosis of anxiety or depression was determined by linkage with the patient's primary care general practice data. Incidence was calculated per 1000 person-years at risk (PYAR). Logistic regression was used to determine risk factors.

**Results:**

Between 2009 and 2018, 179 079 patients met the study criteria and were identified as having a facial scar, and matched to 179 079 controls. The incidence of anxiety in the facial scarring group was 10.05 per 1000 PYAR compared with 7.48 per 1000 PYAR for controls. The incidence of depression in the facial scarring group was 16.28 per 1000 PYAR compared with 9.56 per 1000 PYAR for controls. Age at the time of scarring, previous history of anxiety or depression, female gender, socioeconomic status and classification of scarring increased the risk of both anxiety disorders and depression.

**Conclusions:**

There is a high burden of anxiety disorders and depression in this patient group. Risk of these mental health disorders is very much determined by factors apparent at the time of injury, supporting the need for psychological support.

Facial scarring is broadly classified as either congenital or acquired, occurring across all patient demographics. Although the understanding of wound healing processes and surgical techniques continue to evolve, effective prevention strategies and post-surgical management of facial scarring remains limited.^1^ To date, research has largely focused on the biological mechanisms of scar formation, whereas the incidence of anxiety and depression have been neglected in the literature.^2^

In addition to its physical function, the face is essential for social interaction and has long been considered the most important feature in formulating our perception of identity.^3^ Pressure within modern society to conform to a ‘perfect’ appearance is significant, with stigmatisation reinforced from multiple aspects of society.^4^ Within popular culture, characters from films such as *The Lion King's* ‘Scar’ and *The Dark Knight's* Joker equate scarring with evil, and society's obsession with appearance has been a further mechanism to devalue and marginalise those with facial scarring.^5^

Research has demonstrated that people living with visible differences face significant psychological and social challenges. In addition to having a negative effect on body image, facial scarring can lead to a preoccupation with appearance, loss of confidence and feelings of anger.^4,6–8^ Managing stigmatising reactions from others, such as avoidance and staring, can lead to social avoidance and isolation.^4,9^ Consequently, patients may be vulnerable to developing mental health conditions, such as anxiety disorders and depression.^4^

Anxiety is defined by a pathological worry or dread that undermines normal function, whereas depression is characterised by low mood and anhedonia.^10^ Left untreated, both are common causes of disability with a broad impact on morbidity and mortality. Symptoms of anxiety and depression are linked with increased health costs, influence patient adherence with healthcare, substance misuse, unemployment and poor educational attainment.^11^

## What is known within the literature

Although there is extensive literature demonstrating the psychosocial implications of scarring, the true incidence of anxiety disorders and depression within this cohort have not been well studied. A systematic review and meta-analysis of 21 studies established a pooled prevalence of 26.1% for anxiety and 21.4% for depression.^12^

Of the 21 studies, only eight investigated associated risk factors of anxiety disorders and depression. With regards to aetiology, scars caused by assault were more likely to lead to anxiety disorders and depression than accidental injuries. Female patients had higher risk of an anxiety disorder, but no association was observed between gender and depression. Although numerous studies describe the consequences of the age at the time of facial scarring and its effects on both altered body image and social challenges faced, no study has established an altered risk of either pathology with age. An increased risk was observed in patients with a past history of anxiety disorders or depression. Despite the fact that increased deprivation is an established risk factor for anxiety disorders and depression within the general population, this was not investigated in any of the papers of this study.

The literature in this review had a number of limitations. First, small sample sizes were common; the largest study identified in this review was a prospective case series of 336 participants.^13^ The majority of studies focused on scars caused by acute injury only, limiting their generalisability to the wider scarring population. A high level of bias within the studies was reported because of high attrition rates and inconsistent reporting of results. Finally, follow-up was limited to mostly a year following the scarring event.

## The importance of increasing knowledge of this subject

Knowledge of the incidence of both anxiety disorders and depression within this cohort is essential for practising clinicians for two main reasons. This aspect of facial scarring is often overlooked by services primarily concerned with physical health, leading to suboptimal care.^14^ In a survey of patients with visible differences conducted by the charity Changing Faces, 40% of respondents felt that healthcare professionals did not recognise the psychosocial impact of scarring.^15^

Second, research has demonstrated that patients with psychiatric comorbidities are more likely to seek cosmetic surgery. In a recent study of over a million participants, patients with anxiety were three times more likely to seek reconstructive surgery than controls; patients with depression were twice as likely.^16^ Furthermore, patients with psychiatric comorbidities had a greater risk of developing complications such as infection. This finding reflects those in the wider surgical community, with increased complications found in general surgery, orthopaedic surgery and cardiac surgery.^17,18^ Explanations for this observation include the underlying physiology of the psychiatric disorders and other comorbidities such as substance misuse and social factors.

The objective of this study was to establish the incidence of anxiety and depression in patients with facial scarring, and to compare this with a control population. A secondary objective was to identify risk factors in this group for both anxiety and depression, to help identify those most at risk.

## Method

In this matched cohort study, anonymised individual-level, population-scale, linkable primary and secondary care National Health Service (NHS) data and national administrative data for 2009–2018 in Wales, UK (population of approximately 3.1 million), were analysed within the Secure Anonymised Information Linkage (SAIL) Databank.^19,20^

### Study population

#### Facial scarring group

All patients that had a facial scar from any aetiology between 2009 and 2018 were included in the cohort. Traumatic, acute facial injuries were identified from the Emergency Department Dataset (EDDS), using diagnostic codes for wounds to the face. Patients that had facial surgery of any kind were identified with the Office of Population, Censuses and Surveys Classification of Interventions and Procedures (OPCS-4) codes in the Patient Episode Database for Wales (PEDW). Patients that had conditions leading to facial scarring and deformities (e.g. congenital conditions and skin malignancies) were identified with ICD-10 codes in the PEDW. All diagnostic and procedural codes were independently assessed by J.A.G.G. and T.D.D. (Residents in plastic surgery). Patients that underwent elective procedures or had traumatic events that would cause facial scarring were assumed to have facial scars.

#### Classification of facial scarring

Patients were classified into one of seven categories based on the underlying aetiology of facial scarring, as indicated by their diagnostic code. Acute injuries were classified as caused by an accident, assault, self-harm or cause unknown as recorded in the EDDS. Scars from elective procedures were classified according to their ICD-10 code as follows: benign skin conditions, congenital abnormalities or malignancy.

#### Matched controls

Controls were identified from the Welsh Demographic Service Dataset. They were matched to the facial scarring cohort based on the following demographic variables: socioeconomic status, gender and age at the time of scarring. We aimed to have one control for each case in the study. Cases that were not matched to controls were excluded from the analysis. Socioeconomic status was measured with the Welsh Index of Multiple Deprivation version 2011, the official measure of socioeconomic status by the Welsh Government.^21^ Patients are assigned to one of five quintiles based on their Lower-layer Super Output Area (version 2001) of residence (population approximately 1500), with quintile 1 being the lowest socioeconomic status and 5 being the highest.

### Outcome

The primary outcomes were the development of an anxiety disorder or depression during the study period. Patients were enrolled in the cohort from the time of facial scarring until death, the development of either anxiety or depression, or the end of the study (April 2019). A diagnosis was established from the primary care Welsh Longitudinal General Practice (WLGP) data, as recorded during consultations with patients in general practitioner (GP) records, using Read codes that have been previously validated.^22^ The same Read codes were also used to establish whether patients had a past history of anxiety or depression before their scarring episode.

As the diagnoses of anxiety and depression were based on GP records, patients not enrolled with a general practice contributing data to the SAIL Databank were excluded. The SAIL Databank holds data on approximately 80% of general practices around Wales. Patients diagnosed with anxiety and depression in the year before their scarring event were also excluded, alongside their matched control. A history of an anxiety disorder or depression was defined as a diagnosis of either anxiety or depression, as recorded in the WLGP, more than 1 year before the facial scarring date.

### Ethical approval

The data used in this study are available in the SAIL Databank at Swansea University, Swansea, UK. All proposals to use SAIL data are subject to review by an independent Information Governance Review Panel (IGRP). The IGRP gives careful consideration to each project to ensure proper and appropriate use of SAIL data. When access has been approved, it is gained through a privacy-protecting safe haven and remote access system referred to as the SAIL Gateway. SAIL has established an application process to be followed by anyone who would like to access data via SAIL (https://www.saildatabank.com/application-process).

This work uses data provided by patients and collected by the NHS as part of their care and support. We would also like to acknowledge all data providers who make anonymised data available for research. Approval for this project was obtained from the IGRP under project number 0651.

### Statistical analysis

Baseline characteristics were described using appropriate descriptive statistics. Incidence rates of anxiety disorders and depression were calculated for the entire cohort, and each category of facial scarring with person-years at risk (PYAR) as the denominator. Baseline characteristics were assessed at the date of facial scarring.

Binary logistic regression was used to determine the association between the aetiology of facial scarring and the risk of developing anxiety or depression at 1 year and any point up to 9 years after the scarring event. Initially, this was performed as a univariate analysis to determine risk factors. The following variables were included in this analysis; gender, previous history of anxiety or depression, age at facial scarring and socioeconomic status. Multivariate analysis was then performed, using the risk factors identified through univariate analysis. All data were analysed with IBM SPSS Statistics for Windows (IBM Corp., released 2017, version 25.0; Armonk, New York, USA). Statistical significance was assumed with a *P* < 0.05.

## Results

**Fig. 1:**
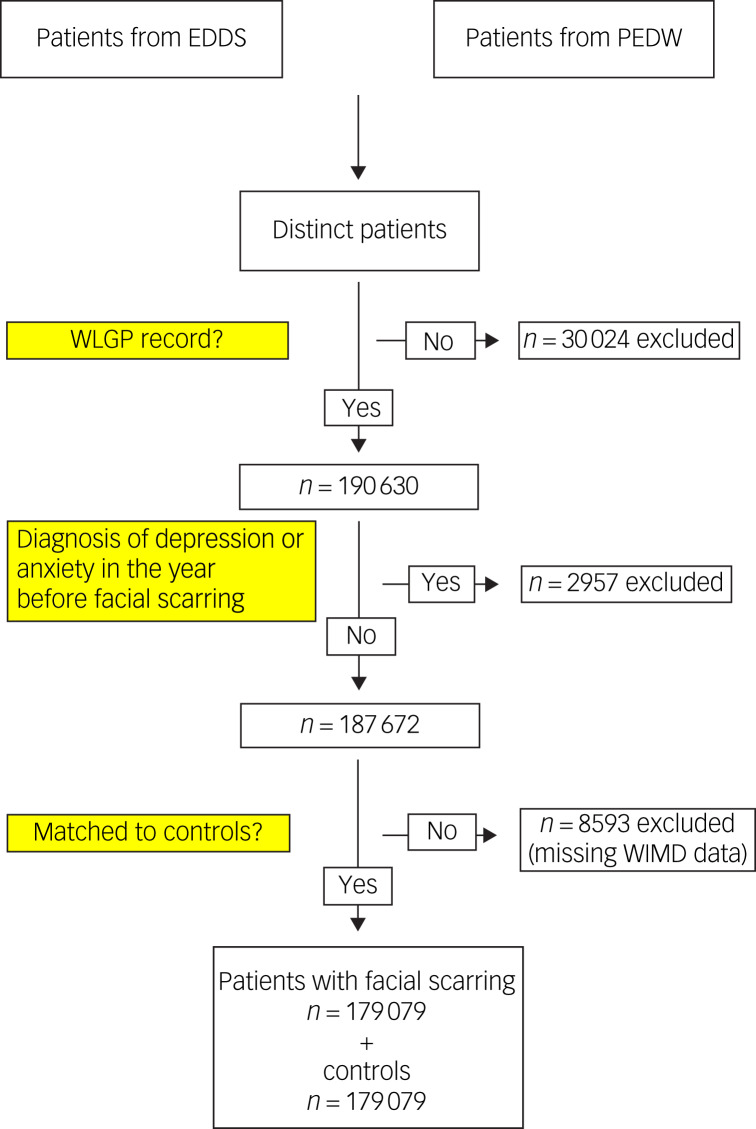
Cohort identification. EDDS, Emergency Department Dataset; PEDW, Patient Episode Database for Wales; WIMD, Welsh Index of Multiple Deprivation; WLGP, Welsh Longitudinal General Practice.

During the study period, a total of 220 654 patients were identified as having sustained a facial scar. Of these, a total of 179 079 patients (81.1%) both met the study criteria and were successfully matched to controls (Fig. 1).

Patient demographics for those in the facial scarring cohort are detailed in Table 1. The facial scarring cohort were exposed to 866 549 PYAR. The median duration of follow-up was 4.7 years (interquartile range 2.3–7.3).

**Table 1:** Patient demographics

Characteristic	Total (*N* = 179 079)	Benign (*n* = 38 969)	Congenital (*n* = 828)	Malignancy (*n* = 21 187)	Accidental injury (*n* = 43 084)	Assault (*n* = 9261)	Self-harm (*n* = 71)	Trauma, aetiology unknown (*n* = 65 679)	*P*-value
Median age (interquartile range), years	38.7 (13.0–68.0)	56.8 (37.9–71.7)	0 (0–0)	74.3 (65.2–82.0)	19.9 (5.0–50.4)	24.3 (17.2–38.1)	28.3 (21.3–41.0)	24.4 (6.6–52.9)	0.00
Age group, *n* (%), years									
0–9	41 083 (22.9)	2095 (5.4)	828 (100)	23 (0.1)	16 742 (38.9)	1500 (16.2)	<5 (2.0)	19 894 (30.2)	0.00
10–19	17 277 (9.6)	2116 (5.4)	0	27 (0.1)	5077 (11.8)	1809 (19.5)	14 (19.7)	8234 (12.5)	
20–29	19 100 (10.7)	2759 (7.1)	0	106 (0.5)	4390 (10.2)	2528 (27.3)	25 (35.2)	9292 (14.1)	
30–39	13 810 (7.7)	3521 (9.0)	0	298 (1.4)	3021 (7.0)	1311 (14.2)	13 (18.2)	5646 (8.6)	
40–49	14 687 (8.2)	4965 (12.7)	0	938 (4.4)	2994 (6.9)	938 (10.1)	10 (14.1)	4842 (7.4)	
50–59	15 061 (8.4)	5818 (14.9)	0	2087 (9.9)	2572 (6.0)	530 (5.7)	<5 (2.0)	4050 (6.2)	
60–69	16 847 (99.4)	6730 (17.3)	0	4395 (20.7)	2298 (95.3)	228 (2.5)	<5 (2.0)	3195 (4.9)	
70–79	19 232 (10.7)	6389 (16.4)	0	6655 (31.4)	2401 (5.6)	170 (1.8)	<5 (2.0)	3616 (5.5)	
80–89	17 138 (9.6)	3924 (10.1)	0	5538 (26.1)	2614 (6.1)	186 (2.0)	0 (0.0)	4876 (7.4)	
≥90	4844 (2.7)	652 (1.7)	0	1120 (5.3)	975 (2.3)	61 (0.6)	<5 (2.0)	2034 (3.1)	
Gender, *n* (%)									
Male	107 379 (60.0)	19 791 (50.8)	447 (54.0)	12 266 (57.9)	27 931 (64.8)	6631 (71.6)	50 (70.4)	40 263 (61.3)	0.00
Female	71 700 (40.0)	19 178 (49.2)	381 (46.0)	8921 (42.1)	15 153 (35.2)	2630 (28.4)	21 (29.6)	25 416 (38.7)	
WIMD 2011 quintile, *n* (%)									
1 (Most deprived)	43 221 (24.1)	7600 (19.5)	226 (27.3)	3319 (15.7)	11 222 (26.0)	3128 (33.8)	23 (32.4)	17 703 (27.0)	0.00
2	34 926 (19.5)	7532 (19.3)	174 (21.0)	3609 (17.0)	9311 (21.6)	1653 (17.8)	21 (29.6)	12 626 (19.2)	
3	33 891 (18.9)	7956 (20.4)	154 (18.6)	4285 (20.2)	8341 (19.4)	1295 (14.0)	12 (16.9)	11 848 (18.0)	
4	30 686 (17.1)	7449 (19.1)	112 (13.5)	4247 (20.0)	7270 (16.9)	1299 (14.0)	10 (14.1)	10 299 (15.7)	
5 (Least deprived)	36 355 (20.3)	8432 (21.6)	162 (19.6)	5727 (27.0)	6940 (16.1)	1886 (20.4)	5 (7.0)	13 203 (20.1)	
Person-years follow-up	866 549.30	188 738.30	4187	90 115	219 577	59 324.00	295.5	394 311.90	
Past history of affective disorder, *n* (%)	39 544 (19.9)	11 484 (29.5)	0 (0.0)	5520 (26.1)	6808 (15.8)	2225 (24.0)	38 (53.5)	13 478 (20.5)	0.00

WIMD, Welsh Index of Multiple Deprivation.

The control cohort contributed to 943 168.90 PYAR. The median duration of follow-up was 5.4 years (interquartile range 2.9–7.7).

### Anxiety disorders

#### Incidence

During the study period, 15 865 (4.4%) patients developed an anxiety disorder: 9095 patients in the facial scarring group (10.05 per 1000 PYAR; 5.1%) and 6770 patients in the control group (7.48 per 1000 PYAR; 3.8%).

#### Identifying risk factors

**Fig. 2:**
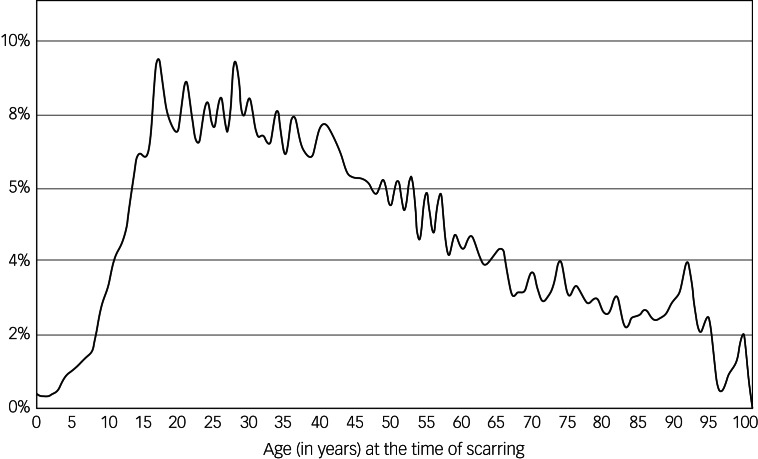
The association of risk of developing anxiety and age at the time of facial scarring.

Univariate logistic regression was used to assess risk with each of the following categorical variables: socioeconomic status, scarring classification and a history of anxiety or depression. This demonstrated that all of the variables were individually, highly significantly associated with both 1-year and 9-year risk of developing an anxiety disorder. To determine the risk associated with the age at the time of facial scarring, cumulative risk of anxiety was determined for each age and plotted in Fig. 2.

#### Multivariable model

Each of the variables described above were used as categorical factors within the multivariate logistic regression model. Following the results of the univariate analysis, age was used as a categorical variable, dividing age ranges into 5-year categories (Table 2).

**Table 2:** Multivariate risk analysis for anxiety disorders

Variable	1 year		9 years	
	*P-*value	Odds ratio	*P*-value	Odds ratio
		(95% CI)		(95% CI)
Age^a^	<0.001	^a^	<0.001	^a^
Gender				
Male	^b^		^b^	
Female	<0.001	1.60 (1.50–1.71)	<0.001	1.56 (1.50–1.61)
WIMD 2011 quintile				
1 (Most deprived)	^b^		^b^	
2	0.17	Not significant	<0.001	0.88 (0.84–0.93)
3	<0.001	0.78 (0.71–0.86)	<0.001	0.85 (0.80–0.89)
4	<0.001	0.78 (0.71–0.87)	<0.001	0.79 (0.75–0.83)
5 (Least deprived)	<0.001	0.83 (0.75–0.91)	<0.001	0.79 (0.75–0.83)
Past history of anxiety or depression	<0.001	4.05 (3.78–4.34)	<0.001	2.94 (2.83–3.06)
Aetiology				
Control	^b^		^b^	
Benign	0.11	Not significant	0.00	1.08 (1.03–1.14)
Congenital	0.99	Not significant	0.26	Not significant
Malignancy	0.38	Not significant	0.19	Not significant
Accidental injury	0.03	0.88 (0.79–0.99)	0.99	Not significant
Assault	<0.001	1.34 (1.15–1.55)	<0.001	1.52 (1.41–1.65)
Self-harm	0.65	Not significant	0.53	Not significant
Trauma, cause unknown	0.99	Not significant	0.2	Not significant

WIMD, Welsh Index of Multiple Deprivation.

a. Age used as a categorical variable and results displayed in Fig. 2.

b. Reference category.

The greatest risk factors identified through multivariate analysis for 1 year and 9 years post-scarring were past medical history of anxiety or depression and age. Both univariate and multivariate analyses demonstrate a sharp increase in risk during puberty. Following this age, the risk of anxiety had an inverse relationship with age (Fig. 2). Female gender and those from more deprived backgrounds were also strong predictors of risk.

Regarding aetiology, the only cause that predicted risk at 1 year was assault; accidental injury had a reduced risk. At 9 years post-scarring, scars from benign causes and from assault were the only aetiologies that increased risk. The reduced risk in the accidental injury aetiology that was observed at 1 year was not observed.

### Depression

#### Incidence

During the study period, 23 387 (6.5%) patients were diagnosed with depression: 14 730 patients in the facial scarring cohort (16.28 per 1000 PYAR; 8.3%) and 8657 in the control group (9.56 per 1000 PYAR, 4.9%).

#### Identifying risk factors

**Fig. 3:**
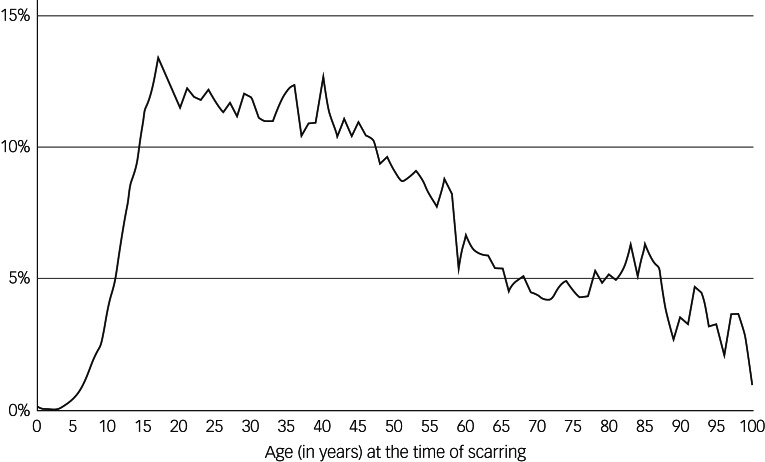
The association of risk of developing depression and age at the time of facial scarring.

Univariate logistic regression was used to assess risk with each of the following categorical variables: socioeconomic status, scarring classification and a history of anxiety or depression. This demonstrated that all of the variables were individually, highly significantly associated with both 1-year and 9-year risk of developing an anxiety disorder. To determine the risk associated with the age at the time of facial scarring, cumulative risk of anxiety was determined for each age and plotted in Fig. 3.

#### Multivariable model

Each of the aforementioned variables were used as factors within the multivariate logistic regression model. Following the results of the univariate analysis, age was used as a categorical variable, dividing age ranges into 5-year categories (Table 3).

**Table 3:** Multivariate risk analysis for depression

Variable	1 Year		9 Years	
	*P-*value	Odds ratio	*P*-value	Odds ratio
		(95% CI)		(95% CI)
Age^a^	0.00		0.00	
Gender				
Male	^b^		^b^	
Female	<0.001	1.44 (1.36–1.52)	<0.001	1.39 (1.35–1.43)
WIMD 2011 quintile				
1 (Most deprived)	^b^		^b^	
2	0.03	0.92 (0.86–0.99)	<0.001	0.9 (0.86–0.94)
3	<0.001	0.86 (0.79–0.93)	<0.001	0.87 (0.83–0.90)
4	<0.001	0.85 (0.78–0.92)	<0.001	0.74 (0.71–0.78)
5 (Least deprived)	<0.001	0.75 (0.70–0.82)	<0.001	0.73 (0.70–0.76)
Past history of anxiety or depression	<0.001	3.98 (3.76–4.20)	0	2.99 (2.90–3.08)
Aetiology				
Control	^b^			
Benign	<0.001	1.15 (1.06–1.24)	<0.001	1.39 (1.33–1.45)
Congenital	0.93	Not significant	<0.001	9.98 (4.53–21.96)
Malignancy	0.25	Not significant	<0.001	1.18 (1.10–1.26)
Accidental injury	<0.001	1.23 (1.12–1.34)	<0.001	1.34 (1.28–1.40)
Assault	<0.001	1.71 (1.53–1.93)	<0.001	2.19 (2.06–2.34)
Self-harm	0.1	Not significant	0.88	Not significant
Trauma, cause unknown	<0.001	1.47 (1.38–1.57)	<0.001	1.41 (1.35–1.46)

WIMD, Welsh Index of Multiple Deprivation.

a. Age used as a categorical variable.

b. Reference category.

The strongest predictors of risk at 1 year and 9 years post-scarring were a past medical history of anxiety or depression and age. In a similar trend to anxiety disorders, risk of depression increased exponentially during puberty. Following this, the risk of depression reduced with increasing age. Female gender and deprivation also increased risk.

At 1 year following the scarring event, assault was the strongest predictor of risk, followed by trauma where the cause was unknown, accidental injury and benign causes. At 9 years, the congenital aetiology had the greatest risk, followed by assault, trauma where the cause was unknown, benign causes and accidental injury.

## Discussion

This is the largest analysis investigating the association of anxiety and depression in patients with facial scarring in the worldwide literature. It has demonstrated that this population has a higher incidence of both anxiety disorders and depression than a cohort of matched controls.

A clear conclusion of this study is that the risk of developing an anxiety disorder or depression is very much affected by risk factors that are apparent at the time of injury. Furthermore, a number of these risk factors were similar for both anxiety disorders and depression. In multivariate analysis, age and a past history of anxiety or depression were the strongest predictors of risk. Age at the time of facial scarring had an interesting relationship with the risk of anxiety and depression: the risk of both increased during adolescence and decreased with advancing age. This finding correlates with psychosocial research in this field, which has demonstrated that during adolescence, appearance plays a crucial role in social belonging and that altered appearance can have significant consequences, such as bullying and poor self-esteem.^4^ During this period, people with visible differences can experience greater levels of teasing and bullying, which can negatively affect self-perception and levels of depressive symptoms.^23^

The risk associated with a history of either an anxiety disorder or depression is of importance for clinicians treating patients with facial scarring. The findings from this study demonstrate the importance of eliciting this information during a focused clinical history, to ensure that support is targeted. Other patient demographic factors, such as female gender and increased level of deprivation, also significantly increased the risk of both diseases, which are established risk factors for anxiety disorders and depression within the general population.^12,24^

An important finding of this study is that there was a high risk of anxiety disorders and depression in patients with facial scarring 9 years after injury. Previous research has been limited to a much shorter follow-up.^12^ This demonstrates that the psychosocial challenges faced by patients with a visible difference are long lasting. Similar to 1-year risk, a number of risk factors were present at the time of diagnosis. Patient factors, such as age, past medical history, deprivation and female gender, contributed to risk for both anxiety disorders and depression.

With respect to aetiology, only scars from assault led to an increased risk of anxiety disorders within the first year. At 9 years, scars from benign causes and assault had an increased risk. Facial scarring from assault has been demonstrated to increase the risk of an anxiety disorder and depression in a number of previous studies,^12,14,25,26^ with patients who experience facial trauma reporting higher rates of substance misuse, post-traumatic stress disorder and stigmatisation, and lower quality of life. Several qualitative studies have reported that scarring might act as a permanent reminder of the assault,^27–30^ and this phenomenon likely leads to continued maladaptive coping strategies and psychological distress.

The fact that some facial scars (congenital, malignancy, accidental injury and trauma where the cause was unknown) did not have increased short-term or long-term risk of developing anxiety could be explained by a number of factors. One explanation is that social anxiety, which is one of the main psychosocial consequences following a visible difference, was not captured in this study, as we mostly focused on generalised anxiety disorders. Furthermore, the diagnosis of an anxiety disorder was determined by GP records, which may underrepresent the true burden of anxiety in this cohort.

With respect to depression, the 1-year risk was high for traumatic aetiologies (assault, accidental injury and trauma where the cause was unknown) and benign scarring. At 9 years, the risk in these categories remained, and an additional increased risk was observed in the congenital and malignant categories. Depression is a prevalent condition in patients with malignancy. This has been attributed to two main pathways: the processes involved with in the biopsychosocial model (biological, psychological and social factors) and the range of specific neuropsychiatric effects of certain cancers and their treatments.^31^

In the literature, a lack of consensus exists as to whether patients with congenital facial disfigurement are more at risk of developing psychological problems. Some studies have demonstrated that adults with congenital facial disfigurement and scarring experience a lower quality of life, lower self-esteem and increased risk of anxiety and depression.^32–34^ However, other studies report no significant increase of psychological problems in this group.^6,35–37^ Our findings must be reviewed with caution as patients entered the study at birth; therefore, the findings are limited to the early years of life.

The self-harm cohort did not have an increased risk of an anxiety disorder or depression. These findings should be taken with caution as the low numbers in this cohort may not truly represent this population.

### Strengths and limitations

A strength of this study is the analysis of large, population-level, routinely collected data, giving large sample sizes. The results reflect the presentation to primary care and the recognition and treatment of anxiety and depression by GPs. Although this method does not carry the attrition bias often seen when using patient-reported outcome measures, it has several limitations. First, electronic medical records can be inaccurate and incomplete. Second, diagnosis is made by a primary care physician and not by a mental health professional, which may be inaccurate. Finally, this method does not capture individuals who do not present to their GP or with whom anxiety and depression are discussed but not recorded.

Patients were excluded from the study when diagnostic and operative codes could not confirm a diagnosis of scarring in any of the data sources. Consequently, the study will have excluded a number of patients with facial scars; however, the large numbers that were included provide a significant level of confidence in our findings.

One limitation of this study is the lack of detail on the level of scar severity; however, this may have limited importance as it is not the objective severity of deformity, but rather the patient's level of satisfaction with their appearance, that has a greater influence on psychological well-being.^35,38^

A limitation of all population-based studies using routinely collected data is incomplete control of confounding, resulting from data that are not specified, incompletely captured or misclassified; namely, aetiology of trauma with cause unknown (relating to either data not being recorded or patients withholding the information).

### Clinical significance of findings

This study demonstrates the higher burden of anxiety disorders and depression in patients with facial scarring. Furthermore, risk factors for developing anxiety disorders and depression are present at the time of presentation to healthcare services. Clinicians from across specialties (surgery, emergency medicine, oncology, dermatology, primary care and paediatrics) should be alert to the possibility of anxiety and depression in those with facial scarring. They should elicit other symptoms, such as insomnia, low mood, anhedonia and suicidal thoughts, and follow National Institute for Health and Care Excellence guidance for the identification (which includes the use of two screening questions) and management of these conditions (which may include sign-posting or referral to other specialities).^39^

At present, psychological care is reactive rather than preventative. This is especially true of patients that sustain scarring from acute facial injuries, who often are treated either in the emergency department or by surgeons who are not trained to screen for or treat anxiety or depression. Furthermore, these patients often do not receive routine follow-up. The findings of this study demonstrate the need for greater access to specialist psychosocial support.

## Supporting information

## Data Availability

The data used in this study are available in the SAIL Databank at Swansea University, Swansea, UK, but as restrictions apply, they are not publicly available. All proposals to use SAIL data are subject to review by an independent Information Governance Review Panel (IGRP). Before any data can be accessed, approval must be given by the IGRP. The IGRP carefully considers each project to ensure the proper and appropriate use of SAIL data. When access has been granted, it is gained through a privacy-protecting trusted research environment (TRE) and remote access system referred to as the SAIL Gateway. SAIL has established an application process to be followed by anyone who would like to access data via SAIL at https://www.saildatabank.com/application-process.
